# Associations between peripheral thyroid sensitivity and all-cause and cardiovascular mortality in the US adults with metabolic syndrome

**DOI:** 10.3389/fmed.2024.1460811

**Published:** 2024-09-11

**Authors:** Qin Deng, Juan Deng, Xiaoyuan Wei, Lu Shen, Jing Chen, Ke Bi

**Affiliations:** ^1^Department of Breast and Thyroid Surgery, The Second Affiliated Hospital of Chongqing Medical University, Chongqing, China; ^2^Cancer Center, West China Hospital, Sichuan University, Chengdu, China; ^3^Department of Emergency, The Second Affiliated Hospital of Chongqing Medical University, Chongqing, China

**Keywords:** metabolic syndrome, peripheral thyroid sensitivity, cohort study, all-cause mortality, cardiovascular mortality

## Abstract

**Background:**

The relationship between peripheral sensitivity to thyroid hormones, as indicated by the ratio of free triiodothyronine (fT3) to free thyroxine (fT4) (fT3/fT4), and the prognosis of metabolic syndrome (MetS) remains unclear.

**Methods:**

This study utilized data from the National Health and Nutrition Examination Survey (NHANES) conducted between 2007 and 2012. MetS was defined based on the criteria established by the National Cholesterol Education Program Adult Treatment Panel III (NCEP-ATP III). Kaplan–Meier survival curves, restricted cubic spline (RCS) analysis, and Cox proportional hazards models were employed to investigate the association between peripheral thyroid sensitivity and mortality outcomes among adults with MetS.

**Results:**

A total of 3,101 adult participants (1,594 males and 1,507 females; median age: 52.00 years) with MetS were included in the analysis. Multivariate Cox regression analysis revealed that elevated levels of fT4 were positively associated with increased risks of both all-cause and cardiovascular mortality in the MetS population [_adjusted_hazard ratio (aHR): 2.74, 95% confidence interval (CI): 1.94–3.87, *p* < 0.001 for all-cause mortality; aHR: 3.93, 95% CI: 2.07–7.45, *p* < 0.001 for cardiovascular mortality]. Conversely, higher levels of fT3 and the fT3/fT4 ratio were found to be protective factors, reducing the mortality risk in the MetS population (fT3: aHR: 0.76, 95% CI: 0.57–0.99, *p* = 0.046 for all-cause mortality; fT3/fT4 ratio: aHR: 0.75, 95% CI: 0.67–0.85, *p* < 0.001 for all-cause mortality; aHR: 0.66, 95% CI: 0.52–0.83, *p* < 0.001 for cardiovascular mortality). The fT3/fT4 ratio exhibited a nonlinear association with all-cause mortality, but a linear and inverse association with cardiovascular mortality.

**Conclusion:**

The findings of this study suggest that higher peripheral thyroid sensitivity, as indicated by the fT3/fT4 ratio, may be associated with reduced mortality risks among adults with MetS. Further research is warranted to validate these associations.

## Introduction

Metabolic syndrome (MetS), defined by a cluster of metabolic risk factors, is recognized as a significant global public health challenge due to its rising prevalence, affecting nearly 25% of the global population (1–3). In the US, the prevalence of MetS exceeds 30%, with a higher incidence observed among older adults (4, 5). Compelling evidence indicates that MetS increases the short- and long-term risks of systemic metabolic diseases and premature mortality (6). Tailoring effective management strategies for this population continues to be a significant challenge. Thus, identifying additional prognostic factors for the MetS population could be instrumental in reducing the burden of adverse clinical outcomes associated with MetS and improving life expectancy (1, 3, 7).

Of note, the thyroid hormone plays a pivotal role in systemic metabolic regulation (8, 9). The free triiodothyronine (fT3), free thyroxin (fT4), and thyroid stimulating hormone (TSH) are the representative indicators in this regulation process (8, 9). Currently, the association between thyroid dysfunction and pathophysiological changes in lipid profiles and the cardiovascular system has been documented (10–12). Hypothyroidism or reduced peripheral thyroid sensitivity can lead to impaired energy expenditure, increased lipid accumulation, and insulin resistance, thereby promoting cardiometabolic dysfunction (13–15). Notably, several studies have identified peripheral sensitivity to thyroid hormones, as measured by the fT3/fT4 ratio, as a potentially optimal indicator for assessing outcomes related to cardiometabolic diseases from the perspective of thyroid function (16–19). Peripheral thyroid sensitivity reflects the responsiveness of peripheral tissues to circulating thyroid hormones, which influence key systemic metabolic processes (20, 21). Altered peripheral thyroid sensitivity may contribute to metabolic dysregulation, playing a role in conditions such as obesity and insulin resistance (22–24). There is also evidence suggesting a significant association between the fT3/fT4 ratio and the occurrence of MetS (15, 25). Specifically, impaired peripheral thyroid sensitivity may affect the distribution of adipose tissue and muscle, further influencing the risk of MetS and its components (26, 27). However, limited research has explored the prognostic impact of peripheral thyroid sensitivity in individuals with MetS, leaving its potential role as a surrogate marker for monitoring this population unclear.

To address these research gaps, the present study aims to investigate the prognostic value of peripheral thyroid sensitivity, especially in terms of fT3/fT4 ratio, in predicting all-cause and cardiovascular mortality among the US adults with MetS, using data from a representative, population-based cohort. This study is one of the few that focuses on the clinical outcomes of the MetS population, examining the prognostic links between peripheral thyroid sensitivity and MetS.

## Materials and methods

### Data source

The study was conducted based on three cycles of interviews between 2007 and 2012 years. Data was derived from the National Health and Nutrition Examination Survey (NHANES) database, encompassing recordings of thyroid function examinations. The NHANES database systematically gathered nationally representative health-related data on the general non-institutionalized US population, utilizing a stratified, multistage probability sampling design (28). Detailed descriptions of NHANES can be found on the official website.^1^ We reported this study following the reporting of observational studies in epidemiology (STROBE) criteria (29).

### Population selection

**FIGURE 1:**
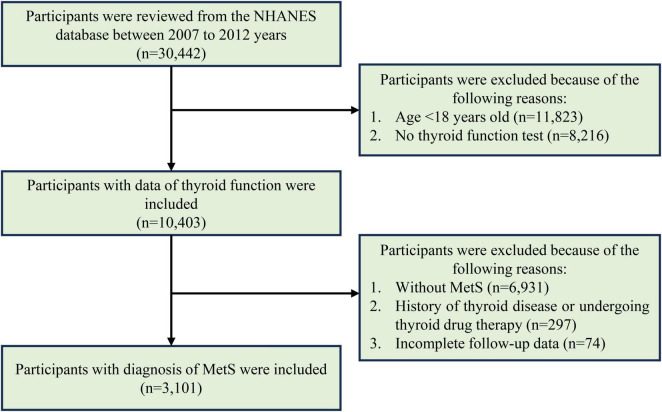
The flowchart for participants’ selection in evaluating the association between peripheral thyroid sensitivity and mortality outcomes of adults with MetS.

To assess the association between peripheral thyroid sensitivity and all-cause as well as cardiovascular mortality in the MetS population, only participants diagnosed with MetS at the time of the interview were included in the study. Participants were excluded if they lacked thyroid function test results, had incomplete follow-up data, had a self-reported history of thyroid diseases, or undergoing thyroid drug therapy. However, individuals without self-reported thyroid disease but with abnormal thyroid antibodies were included, as these may reflect subclinical or early-stage autoimmune thyroid conditions rather than overt thyroid disease. The participants’ selection process is shown in Figure 1.

### Definition of MetS

The definition of MetS was based on the [National Cholesterol Education Program III (NCEP III): National Cholesterol Education Program Adult Treatment Panel III] (3, 30). Participants who met three or more of the following criteria: (1) the fasting plasma glucose (FPG) > 100 mg/dL or drug treatment for diabetes mellitus; (2) high-density lipoprotein cholesterol (HDL-c) < 50 mg/dL in females, < 40 mg/dL in males or drug treatment for reduced HDL-c; (3) Plasma triglycerides > 150 mg/dL or drug treatment for raised triglycerides; (4) Waist circumference > 88 cm in women or > 102 cm in men; (5) Blood pressure > 130/85 mmHg or drug treatment for raised blood pressure.

### Thyroid-related indicators assessment

Five thyroid-related indicators, including fT3, fT4, the fT3/fT4 ratio, TSH, thyroglobulin antibody (TgAb), and thyroid peroxidase antibody (TPOAb), were collected and calculated. Thyroid function was assessed through peripheral blood tests using automated hematology analysis during each interview cycle. Serum levels of thyroid function indicators were analyzed as continuous variables or categorized into quartiles for each indicator level.

### Covariates assessment

The selection of study covariates in the evaluation of the association between thyroid-related indicators and prognostic of the MetS population was based on previous literature (1, 3, 30). Demographic factors included sex (classified as male or female, considering sex differences in thyroid function and metabolic outcomes), age (older age was associated with higher mortality, potentially confounding the relationship between peripheral thyroid sensitivity and mortality), and race/ethnicity (classified as Hispanic, non-Hispanic White, non-Hispanic Black, and other, accounting for ethnic and racial differences in thyroid function and mortality risk). Socioeconomic factors included marital status (unmarried, married, or living with a partner), educational level (≤ high school, college, and > college), and family income poverty ratio (< 1.3, 1.3–3.5, and > 3.5, reflecting access to healthcare and overall health) were also controlled. Lifestyle factors including smoking status (never, former, current) and alcohol use (never, former, current), along with common comorbidities including history of cancer, cardiovascular disease (CVD), chronic kidney disease (CKD), and body mass index (BMI), were considered due to their potential impact on thyroid function and mortality. Additionally, thyroid-related indicators such as TSH, TgAb, and TPOAb were included in the analysis to account for their influence on fT3, fT4, and the fT3/fT4 ratio.

### Outcome measurements

The primary mortality outcome considered in our study was all-cause and cardiovascular mortality of the MetS population. The mortality data for the follow-up population were obtained from the NHANES public-use linked mortality file as of 31 December 2019 (31). Additionally, the ICD-10 (International Statistical Classification of Diseases, 10th revision) was applied to identify the cause-specific mortality.

### Statistical analysis

According to the NHANES analytic tutorials for weights in making estimates that were representative of the US civilian non-institutionalized population,^2^ all analyses in this study incorporated sample weights, clustering, and stratification to estimate appropriate variance and ensure national representation of US population with MetS. The demographic characteristics between survivors and non-survivors were compared using variance analysis for continuous variables (reported as median with 1st quartile and 3rd quartile) and the Chi-Squared test for categorical variables. Univariate and multivariable Cox proportional hazard models were used to estimate the association of fT3 and fT4 as well as the fT3/fT4 ratio with all-cause, cardiovascular mortality of the MetS population. Model 1 referred to the unadjusted analysis. Model 2 referred to adjustments for age, sex, and race. As for model 3, we accounted for age, sex, race, marriage status, educational level, family income-poverty ratio, smoking status, cancer, CKD, CVD, BMI, and serum levels of TSH, TgAb, and TPOAb.

Kaplan–Meier (KM) curves were used to show the different survival probabilities among the MetS population with varied quartiles of fT3, fT4, and fT3/fT4 ratio. To assess the non-linear association between peripheral thyroid sensitivity and both all-cause and cardiovascular mortality in the MetS population, we employed restricted cubic splines (RCS). In this study, the fT3/fT4 ratio was treated as a continuous variable, and four pre-specified knots were used based on its distribution. The RCS model was then used to explore the relationship between the fT3/fT4 ratio and mortality outcomes. The selection of the number and location of these knots was carefully considered to ensure model robustness while avoiding overfitting. Variables with missing data were interpolated using multiple interpolations (32). The concordance index (C-index) was used to evaluate the predictive ability of the fT3, fT4, and the fT3/fT4 ratio in predicting the mortality outcomes of adults with MetS.

The sensitivity analyses were conducted to validate the main findings. First, we excluded participants who died within two years after the interview. Second, we restricted the study population to the initial two cycles of surveys. Last, the fT3/fT4 ratio was calculated as categorical variables to reevaluate the association between the peripheral thyroid sensitivity with all-cause and cardiovascular mortality of the MetS population.

All statistical analyses were conducted using the R software (version 4.2.3).^3^ The two-tailed *p* < 0.05 was considered statistically significant.

## Results

### The baseline feature of the adults with MetS

A total of 3,101 participants were included in this study, with a median age of 52.00 years old and males counted for a slightly higher proportion (1,594 cases, 51.8%). The median follow-up time was 132 months. A majority of the study population was non-Hispanic white (1,435 cases, 71.1%) and had an education level below the college (1,866 cases, 51.1%). The frequencies of comorbidities including cancer, CKD, and CVD were 11.7% (363 cases), 14.8% (639 cases), and 9.7% (395 cases), respectively. The median serum levels of fT3, fT4, TSH, TgAb, and TPOAb were 3.20 pg/mL, 0.80 pg/mL, 1.76 mIU/L, 0.60 IU/mL, and 0.60 IU/mL, respectively. The median fT3/fT4 ratio was 4.14. Compared with survivors, non-survivors presented characteristics of older age, males, lower socioeconomic status lower serum levels of fT3 and fT3/fT4 ratio, and a higher rate of comorbidities (All *p* < 0.05). The detailed information is summarized in Table 1. Additionally, compared with participants without CVD, those with a history of CVD had lower peripheral thyroid sensitivity (*p* < 0.001) and presented a significantly higher all-cause as well as cardiovascular mortality (*p* < 0.001) (Table 2).

**TABLE 1:** The demographic characteristics of the adults with MetS in this study.

Variable	Total (*n* = 3,101)	Survivors (*n* = 2,494)	Non-survivors (*n* = 607)	*p*
Age	52.00 (40.00, 63.00)	49.02 (38.00, 59.00)	69.00 (60.00, 78.00)	**< 0.001**
BMI	31.40 (28.12, 35.80)	31.66 (28.38, 36.01)	30.17 (26.77, 34.59)	**< 0.001**
fT3	3.20 (2.97, 3.40)	3.20 (3.00, 3.41)	3.00 (2.80, 3.24)	**< 0.001**
fT4	0.80 (0.70, 0.87)	0.80 (0.70, 0.84)	0.80 (0.70, 0.90)	**< 0.001**
fT3/fT4 ratio	4.14 (3.63, 4.71)	4.22 (3.71, 4.71)	3.78 (3.17, 4.35)	**< 0.001**
TSH	1.76 (1.19, 2.54)	1.75 (1.19, 2.49)	1.79 (1.22, 2.76)	0.062
TgAb	0.60 (0.60, 0.60)	0.60 (0.60, 0.60)	0.60 (0.60, 0.60)	0.617
TPOAb	0.60 (0.30, 1.59)	0.60 (0.30, 1.50)	0.60 (0.30, 1.70)	0.418
**Sex**				**0.031**
Male	1,594 (51.8)	1,236 (51.2)	358 (57.3)	
Female	1,507 (48.2)	1,258 (48.8)	249 (42.7)	
**Race**				**< 0.001**
Hispanics	940 (14.0)	842 (16.1)	98 (6.6)	
Non-Hispanics white	1,435 (71.1)	1,065 (68.7)	370 (79.6)	
Non-Hispanics black	573 (9.7)	450 (9.6)	123 (10.6)	
Other	153 (5.2)	137 (5.6)	16 (3.2)	
**Educational level**				**0.003**
≤ High school	1,866 (51.1)	1,458 (49.8)	408 (60.2)	
College	805 (29.7)	671 (30.6)	134 (25.9)	
> College	430 (19.2)	365 (19.6)	65 (13.9)	
**Marital status**				**< 0.001**
Unmarried	1,179 (32.3)	896 (30.2)	283 (40.9)	
Married or living with a partner	1,922 (67.7)	1,598 (69.8)	324 (59.1)	
**Family income poverty ratio**				**< 0.001**
< 1.3	964 (22.2)	761 (21.2)	203 (25.1)	
1.3–3.5	1,383 (40.6)	1,068 (37.6)	315 (55.1)	
> 3.5	754 (37.2)	665 (41.2)	89 (19.8)	
**Cancer**				**< 0.001**
No	2,738 (88.3)	2,265 (90.3)	473 (77.2)	
Yes	363 (11.7)	229 (9.7)	134 (22.8)	
**CKD**				**< 0.001**
No	2,462 (85.2)	2,147 (90.2)	315 (56.7)	
Yes	639 (14.8)	347 (9.8)	292 (43.3)	
**CVD**				**< 0.001**
No	2,706 (90.3)	2,288 (93.2)	418 (70.1)	
Yes	395 (9.7)	206 (6.8)	189 (29.9)	
**Smoking status**				**< 0.001**
Never	1,559 (50.6)	1,327 (53.1)	232 (37.2)	
Current	625 (20.8)	503 (19.8)	122 (23.1)	
Ever	917 (28.6)	664 (27.1)	253 (39.7)	
**Alcohol use**				**< 0.001**
Never	759 (22.3)	533 (20.3)	226 (35.7)	
Current	1,704 (60.0)	1,465 (64.1)	239 (42.2)	
Ever	638 (17.7)	496 (15.6)	142 (22.1)	

(1) For the continuous statistics reported are the sample median with the 1st quartile and 3rd quartile in parentheses. (2) For the categories, statistics reported are the sample frequency with the percentage in parentheses. (3) Bold value means statically significant. fT3, free triiodothyronine; fT4, free thyroxin; BMI, body mass index; TSH, thyroid stimulating hormone; TgAb, thyroglobulin antibodies; TPOAb, thyroid peroxidase antibody; CKD, chronic kidney disease; CVD, cardiovascular disease.

**TABLE 2:** Comparison of thyroid-related indicators between participants with and without a history of CVD.

Variable	Total (*n* = 3,101)	Non-CVD (*n* = 2,706)	CVD (*n* = 395)	*p*
fT3	3.20 (2.97, 3.40)	3.20 (3.00, 3.40)	3.00 (2.79, 3.20)	**< 0.001**
fT4	0.80 (0.70, 0.87)	0.80 (0.70, 0.86)	0.80 (0.70, 0.90)	**0.001**
fT3/fT4 ratio	4.14 (3.63, 4.71)	4.19 (3.67, 4.71)	3.86 (3.25, 4.33)	**< 0.001**
TSH	1.76 (1.19, 2.54)	1.75 (1.19, 2.52)	1.90 (1.29, 2.57)	0.338
TgAb	0.60 (0.60, 0.60)	0.60 (0.60, 0.60)	0.60 (0.60, 0.60)	0.945
TPOAb	0.60 (0.30, 1.59)	0.60 (0.30, 1.50)	0.70 (0.30, 1.60)	0.732
**All-cause mortality**				**< 0.001**
No	2,494 (84.8)	2,288 (87.8)	206 (55.9)	
Yes	607 (15.2)	418 (12.2)	189 (44.1)	
**Cardiovascular mortality**				**< 0.001**
No	2,955 (96.7)	2,625 (97.9)	330 (86.2)	
Yes	146 (3.3)	81 (2.1)	65 (13.8)	

(1) For the continuous statistics reported are the sample median with the 1st quartile and 3rd quartile in parentheses. (2) For the categories, statistics reported are the sample frequency with the percentage in parentheses. (3) Bold value means statically significant. fT3, free triiodothyronine; fT4, free thyroxin; TSH, thyroid stimulating hormone; TgAb, thyroglobulin antibodies; TPOAb, thyroid peroxidase antibody; CVD, cardiovascular disease.

### The non-linear trend between peripheral thyroid sensitivity and mortality of adults with MetS

**FIGURE 2:**
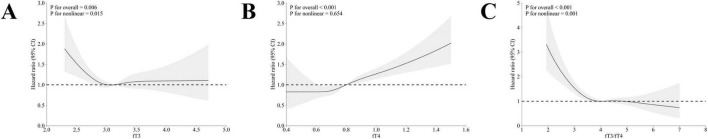
The association between fT3, fT4, and fT3/fT4 ratio with all-cause mortality in adults with MetS by multivariable-adjusted restricted cubic splines. **(A)** The association between fT3 levels and all-cause mortality; **(B)** The association between fT4 levels and all-cause mortality; **(C)** The association between fT3/fT4 ratio levels and all-cause mortality. The dotted lines represent 95% confidence intervals. Spline analyses were adjusted for age, sex, race, marriage status, educational level, family income-poverty ratio, smoking status, alcohol use, history of cancer, chronic kidney disease, cardiovascular disease, BMI, and TSH, TgAb, and TPOAb.

**FIGURE 3:**
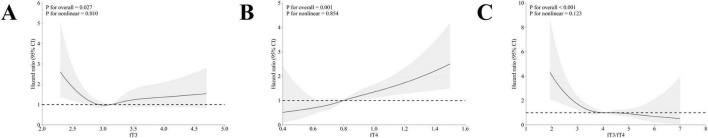
The association between fT3, fT4, and fT3/fT4 ratio with cardiovascular mortality in adults with MetS by multivariable-adjusted restricted cubic splines. **(A)** The association between fT3 levels and cardiovascular mortality; **(B)** The association between fT4 levels and cardiovascular mortality; **(C)** The association between fT3/fT4 ratio levels and cardiovascular mortality. The dotted lines represent 95% confidence intervals. Spline analyses were adjusted for age, sex, race, marriage status, educational level, family income-poverty ratio, smoking status, alcohol use, history of cancer, chronic kidney disease, cardiovascular disease, BMI, and TSH, TgAb, and TPOAb.

The multivariable-adjusted RCS curves showed a non-linear association of fT3 with all-cause mortality (*p* for nonlinearity = 0.015) of the MetS population (Figure 2A), with a cutoff point of 3.2 pg/mL. Alternatively, linear trends were observed between fT4 and all-cause mortality (*p* for overall < 0.001, Figure 2B). Notably, the fT3/fT4 ratio showed non-linear associations with all-cause mortality in the population with MetS, with a cutoff point of 4.1 (*p* for nonlinear = 0.001) (Figure 2C). Meanwhile, a non-linear relationship between the level of fT3 and cardiovascular mortality of adults with MetS was observed (*p* for nonlinear = 0.010) (Figure 3A). However, the linear trends were determined between levels of fT4 and fT3/fT4 ratio with cardiovascular mortality (fT4: *p* for overall = 0.001; fT3/fT4 ratio: *p* for overall < 0.001, respectively) among the adults with MetS (Figures 3B, C).

### Kaplan–Meier curves of the survival patterns in adults with MetS

**FIGURE 4:**
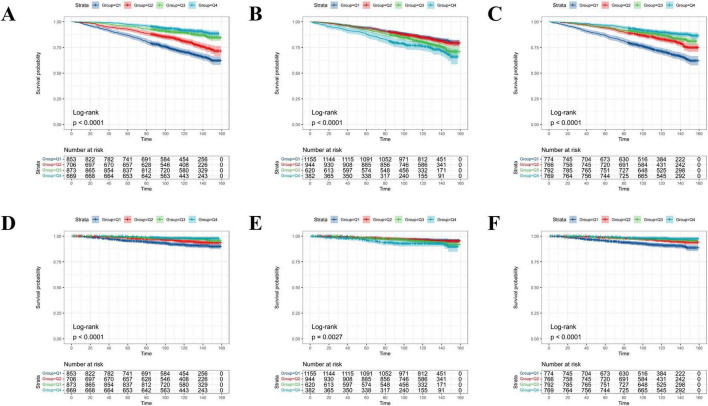
The probability of all-cause and cardiovascular-specific survival over time for adults with MetS stratified by quartile levels of fT3, fT4, and the fT3/fT4 ratio. **(A)** All-cause survival probability stratified by fT3 levels; **(B)** all-cause survival probability stratified by fT4 levels; **(C)** all-cause survival probability stratified by fT3/fT4 ratio; **(D)** cardiovascular survival probability stratified by fT3 levels; **(E)** cardiovascular survival probability stratified by fT4 levels; **(F)** cardiovascular survival probability stratified by fT3/fT4 ratio.

The fT3, fT4, and fT3/fT4 ratios were calculated as quartiles. As shown in Figure 4, the KM curves showed that the MetS population with higher levels of fT3 (3rd and 4th quartiles) showed prolonged survival probabilities than those in lower levels of fT3 (1st and 2nd quartiles) irrespective of the overall (Figure 4A) and cardiovascular specific survivals (Figure 4D). By contrast, the higher serum level of fT4 was negatively associated with the survival probabilities of the MetS population (Figures 4B, E). The survival patterns, particularly the overall survival (Figures 4C, F), became more remarkably different once the fT3/fT4 ratio was used as the classifier.

### Association between peripheral thyroid sensitivity with all-cause mortality

**FIGURE 5:**
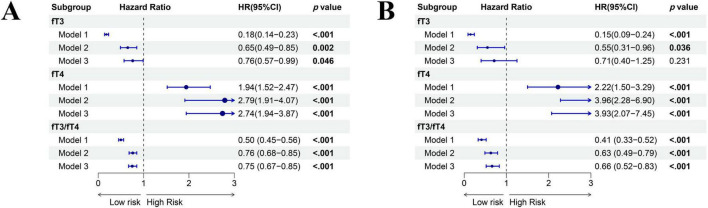
Associations between the levels of fT3, fT4, and fT3/fT4 ratio with mortality outcomes of adults with MetS by three Cox regression analyses models **(A)** The association between levels of fT3, fT4, and fT3/fT4 ratio with all-cause mortality; **(B)** The association between levels of fT3, fT4, and fT3/fT4 ratio with cardiovascular mortality. Results are shown for three Cox regression models: Model 1 (unadjusted), Model 2 (adjusted for age, sex, and race), and Model 3 (fully adjusted for age, sex, race, marital status, educational level, family income-poverty ratio, smoking status, alcohol use, history of cancer, chronic kidney disease, cardiovascular disease, BMI, and levels of TSH, TgAb, and TPOAb).

The Cox proportional hazard model showed that fT3/fT4 ratio was significantly associated with the all-cause mortality of MetS population [Unadjusted: hazard ratio (HR) = 0.50, 95% confidence interval (CI): 0.45–0.56, *p* < 0.001; Less adjusted: HR = 0.76, 95% CI: 0.68–0.85; *p* < 0.001; Full adjusted: HR = 0.75, 95% CI: 0.67–0.85, *p* < 0.001] (Figure 5A). Respectively, fT3 was negatively associated with the all-cause mortality [Unadjusted: HR = 0.18, 95% CI: 0.14–0.23, *p* < 0.001; Less adjusted: HR = 0.65, 95% CI: 0.49–0.85; *p* = 0.002; Full adjusted: HR = 0.76, 95% CI: 0.57–0.99, *p* = 0.046], whereas fT4 was significantly positive associated with the all-cause mortality of MetS population [Unadjusted: HR = 1.94, 95% CI: 1.52–2.47, *p* < 0.001; Less adjusted: HR = 2.79, 95% CI: 1.91–4.07; *p* < 0.001; Full adjusted: HR = 2.74, 95% CI: 1.94–3.87, *p* < 0.001]. The C-index was 0.793 in the fT3/fT4 ratio, 0.664 in fT3, and 0.561 in fT4, respectively (Supplementary Table 1).

### Association between peripheral thyroid sensitivity with cardiovascular mortality

The Cox proportional hazard model showed that the fT3/fT4 ratio was significantly associated with the cardiovascular mortality of the MetS population [Unadjusted: HR = 0.41, 95% CI: 0.33–0.52, *p* < 0.001; Less adjusted: HR = 0.63, 95% CI: 0.49–0.79; *p* < 0.001; Full adjusted: HR = 0.66, 95% CI: 0.52–0.83, *p* < 0.001] (Figure 5B). Regarding the fT3, no significant association was observed in cardiovascular mortality of the MetS population after adjusting covariates [HR = 0.71, 95% CI: 0.40–1.25, *p* = 0.231]. However, fT4 remained significantly associated with the cardiovascular-related mortality of MetS population [Full adjusted: HR = 3.93, 95% CI: 2.07–7.45, *p* < 0.001]. Combined with other characteristics, the C-index was 0.797 in the fT3/fT4 ratio, 0.686 in fT3, and 0.581 in fT4, respectively (Supplementary Table 1).

### Stratified and sensitive analyses

The stratified analyses showed MetS population with clinical characteristics of Hispanics and other races, high educational level and family income poverty ratio, and abnormal antithyroid antibodies showed less sensitivity to the survival prediction value of fT3/fT4 ratio (Supplementary Figures 1, 2). There was only one interaction observed in the family income poverty ratio for cardiovascular mortality (Supplementary Figure 2). Meanwhile, different sensitive analyses were conducted. First, participants who died within 2 years after the interview were excluded. As shown in Supplementary Tables 2, 3, consistent associations were observed in the modified MetS population. Second, we performed the Cox regression analyses in two cycles of surveys settled between 2007 and 2010. As expected, the results also supported the main findings mentioned above (Supplementary Tables 4, 5). Last, the results were still significant once the fT3/fT4 ratio was calculated as the categorical variable (Supplementary Tables 6–9).

## Discussion

In the present study, a significant association was observed between peripheral thyroid sensitivity and both all-cause and cardiovascular mortality among the MetS population. A higher level of fT4 level was positively associated with increased all-cause as well as cardiovascular mortality risks, while fT3 was only negatively associated with all-cause mortality. Additionally, participants with a higher fT3/fT4 ratio, particularly those with a ratio above 4.1, had a lower risk of all-cause and cardiovascular mortality compared to those in the lower ratio group.

Currently, the high prevalence of MetS remains a huge public health issue that affects millions of adults worldwide (1, 3). A series of secondary comorbidities caused by the MetS condition would increase the risk for mortality in later life (1, 3). Individuals with MetS are at a significantly higher risk for both all-cause and cause-specific mortality compared to the non-MetS population (33–36). For instance, one recent large-scale prospective cohort study revealed that the MetS significantly increased the risk of cardiopulmonary morbidity and all-cause mortality at all levels of lung function impairment (35). In one earlier meta-analysis, Watanabe and Kotani (36) demonstrated that MetS significantly predicted CVD morbidity and cardiovascular mortality among the general Japanese population. These findings highlighted the importance of making adequate management of MetS to reduce mortality and improve long-term health outcomes. During the past decades, the association between thyroid dysfunction with the risk for MetS has been determined (37). For instance, Heima et al. (10) observed a positive association between serum levels of TSH with the prevalence of MetS in the Dutch older population (10). This finding was supported in recent studies conducted in other countries (12, 38). Mechanically, the regulations of glucose metabolism, lipid metabolism, and blood pressure by the thyroid hormones were considered to play a pivotal role in MetS (27, 39). Cross-sectional studies revealed the plausible roles of serum levels of fT3 and fT4 in affecting the levels of low-density lipoprotein (LDL-c) and high-density lipoprotein cholesterol (HDL-c) (11, 40, 41). Most recently, the Mendelian randomization (MR) study preliminary revealed the genetical causality between thyroid function and the MetS that higher fT4 levels would reduce the risk of developing MetS (42). As they reported, the fT4 was genetically positively associated with HDL-c, whereas TSH was positively associated with triglyceride. On the other hand, another study from the NHANES suggested even mild resistance to thyroid hormones was also associated with the development of MetS (15).

Nevertheless, regarding the association between thyroid function with mortality in the general population, the conclusions remained debatable. In one earlier study among the US population (*n* = 1,587), Waring et al. (43) did not observe a significant association between thyroid function the all-cause or cause-specific mortalities. By contrast, in another study settled in the Netherlands (*n* = 7,785), low-normal thyroid function levels showed protective effects on older participants without CVD, which would prolong the life expectancies for over 3 years than participants with high-normal thyroid function (44). However, limited studies were available in reevaluating the prognostic role of peripheral sensitivity to thyroid hormones among the MetS population, the group with high cardiometabolic dysfunction burdens. In the current study, we filled this research gap and observed that the fT3/fT4 ratio was remarkably associated with the all-cause mortality of the MetS population, accompanied by distinctive roles in this process. Especially, participants with a high fT3/fT4 ratio showed a lower risk for mortality when compared to those with a low fT3/fT4 ratio. Compared with a single thyroid hormone indicator, the ratio of fT3/fT4 presented superior predictive power in the mortality of the MetS population. Recently, the fT3/fT4 ratio was observed to be a promising predictor in the prognosis of metabolic-related diseases. Namely, low levels of fT3 or high levels of fT4 frequently predicted worse clinical outcomes (19, 45, 46). Some possible explanations might be highlighted. Specifically, low fT3 might be indicative of nonthyroidal systemic illnesses of the participants (47–49). For instance, He et al. (45) observed that participants with decreased fT3/fT4 ratio presented greater levels of systemic inflammatory condition and significantly lower levels of hemoglobin and albumin, which suggested a more severe degree of inflammation and subsequent worse clinical status of this subpopulation (45).

Additionally, the current study determined that the fT3/fT4 ratio was associated with cardiovascular mortality in the MetS population. Consistent with previous studies focused on the general population (50), participants with high levels of fT4 demonstrated an increased risk for cardiovascular mortality. The strong connection between thyroid function and cardiovascular health is well-established, as the cardiovascular system is a major target of thyroid hormones (51). The fT3/fT4 ratio, an indicator of peripheral thyroid hormone sensitivity, has emerged as a potential marker for cardiovascular risk stratification (16). A lower fT3/fT4 ratio might reflect a reduced conversion of T4 to the more biologically active T3, which could be indicative of an adaptive response to chronic diseases (52, 53). This was particularly relevant in the context of MetS, where altered thyroid hormone metabolism could increase cardiovascular risks. Specifically, a lower fT3/fT4 ratio was observed to be associated with increased arterial stiffness, endothelial dysfunction, and impaired lipid metabolism, all of which were critical contributors to cardiovascular mortality (51, 54). Nevertheless, the association between thyroid function and cardiovascular mortality remains inconclusive (55–57). While one earlier study found no significant elevated risks of cardiovascular mortality among populations with subclinical hyper- and hypothyroidism (58), more recent studies demonstrated that abnormal thyroid function was independently associated with increased mortality in both populations with and without cardiovascular disease (56, 59).

Notably, compared to the general US population, our study found that the fT3/fT4 ratio had stronger predictive power for cardiovascular mortality in the MetS population (HR = 0.66 in the MetS population vs. HR = 0.82 in the general US population) (16). These findings, along with previous research, underscored the importance of reevaluating and selectively screening thyroid function in specific populations, particularly those with MetS. Given the bidirectional relationship between thyroid function and MetS (27, 60), standardizing thyroid function management for the MetS population remains a challenge. Therefore, it is crucial to consider preventive strategies for patients with MetS who exhibit a peripheral thyroid resistance profile, including regular monitoring of thyroid hormone levels, lifestyle modifications, and optimizing cardiovascular risk factors through appropriate medical interventions (61, 62). Large randomized clinical trials are needed to provide robust evidence on the cost-benefit of screening thyroid function in preventing cardiovascular events and mortality among the MetS population.

In stratified analysis, the lack of significant prognostic predictive value of the fT3/fT4 ratio for mortality in subgroups such as Hispanics and other ethnicities, individuals with higher education levels, higher family income, and those with abnormal thyroid antibodies might be attributed to several potential factors. In particular, higher socioeconomic status is often associated with better access to healthcare and more effective disease management, potentially reducing the impact of peripheral thyroid sensitivity on mortality (63, 64). Additionally, ethnic differences might influence thyroid hormone dynamics and the optimal normal range of thyroid function, affecting the predictive ability of the fT3/fT4 ratio (63–66). Individuals with abnormal thyroid antibodies might alter thyroid hormone balance, diminishing the relevance of the fT3/fT4 ratio as the mortality predictor (37). Interestingly, the fT3/fT4 ratio showed stronger significance in predicting cardiovascular mortality in the MetS population with a history of CVD. Thyroid hormones directly impact the cardiovascular system by influencing heart rate, vascular resistance, and lipid metabolism (13, 14, 61). In individuals who had a history of CVD, even subtle changes in thyroid function might exacerbate underlying cardiovascular conditions, leading to more pronounced effects on mortality risk. Therefore, the fT3/fT4 ratio may serve as a more sensitive marker for adverse outcomes in MetS patients with pre-existing cardiovascular conditions, reflecting the heightened vulnerability of this subgroup to thyroid hormone imbalances. Further studies are warranted to explore the connection between peripheral thyroid sensitivity and mortality outcomes of MetS in specific subgroups.

Some noteworthy strengths need to be highlighted in the present study. First, to our knowledge, this is only one of few works focusing on the prognostic role of thyroid function in the MetS population within long-term follow-up data. Second, the prospective, population-based study design helps us to determine the robust evidence between the fT3/fT4 ratio and all-cause as well as cardiovascular mortality of the MetS population. Last, the series of sensitive analyses showed consistent associations as we determined in the main findings.

Admittedly, there are some limitations existing in the present study. First, while the present study was conducted based on one large-scale, population-based cohort, the evaluation of thyroid-related parameters was only collected at a single measurement during the interview. However, this might not significantly impair our findings, given that the normal range of thyroid function was considered to be stable over time (67). Moreover, we excluded the participants with a history of thyroid diseases or receiving thyroid drug therapy at initial selection, which would help to reduce the potential impact of drugs on peripheral thyroid sensitivity. Future works focused on trajectories of thyroid function would provide more robust evidence on this topic. Although we controlled for several confounders, including socioeconomic status, common comorbidities, and lifestyle factors, some residual confounders (such as iodine intake, genetic heterogeneity in thyroid hormone resistance, dietary habits, physical activity, exposure to environmental toxins, and mental health) were not available in the dataset used for this study. These factors have been observed to influence thyroid function and mortality risks in the MetS population and might have introduced some degree of residual confounding. In addition, previous studies revealed that the MetS population would present abnormal thyroid function as well, which indicated that potential reverse causality existed between thyroid function and MetS. Last, some specific subpopulations including the MetS concurrent with Hashimoto’s thyroiditis, subclinical hyperthyroidism, or subclinical hypothyroidism, were not further investigated due to the small sample size and events (68). Whether the fT3/fT4 ratio shows a consistently significant association with the mortality outcomes in populations of different races from varied countries is worth exploring in the future. Therefore, designing prospective longitudinal studies and identifying novel gene targets would help to address these shortages in future works.

## Conclusion

In this study, we observed a significant association between peripheral thyroid sensitivity and both all-cause and cardiovascular mortality in adults with MetS. Our findings suggest that peripheral thyroid sensitivity may serve as a valuable predictive marker for mortality outcomes in the MetS population, potentially acting as a surrogate biomarker for long-term monitoring. The findings of this study underscore the importance of incorporating thyroid-related indicators in the routine monitoring of MetS patients, aiding physicians in making more personalized health evaluations and clinical decisions. Future research is needed to further validate these findings.

## Data Availability

The raw data supporting the conclusions of this article will be made available by the authors, without undue reservation.

## Footnotes

1
https://www.cdc.gov/nchs/nhanes/

2
https://wwwn.cdc.gov/nchs/nhanes/tutorials/weighting.aspx

3
https://www.r-project.org/
