# Supplementary material for: Associations between peripheral thyroid sensitivity and all-cause and cardiovascular mortality in the US adults with metabolic syndrome
Source: Front Med (Lausanne). 2024 Sep 11;11:1460811. doi: 10.3389/fmed.2024.1460811 (PMC11422239; doi:10.3389/fmed.2024.1460811)
Supplement: Supplementary file 1 [file Data_Sheet_1.docx]

**Supporting information**

**Associations between Peripheral Thyroid Sensitivity and All-cause and Cardiovascular Mortality in the US Adults with Metabolic Syndrome**

Qin Deng ^1^, Juan Deng ^1^ , Xiaoyuan Wei ^2^, Lu Shen ^1^, Jing Chen ^1^, Ke Bi ^3*^

^1^ Department of Breast and Thyroid Surgery, The Second Affiliated Hospital of Chongqing Medical University, Chongqing, 400010, China.

^2^ Cancer Center, West China Hospital, Sichuan University, Chengdu, China

^3^ Department of Emergency, The Second Affiliated Hospital of Chongqing Medical University, Chongqing, 400010, China.

*Corresponding Authors:

Prof. Ke Bi is to be contacted at the Department of Emergency, The Second Affiliated Hospital of Chongqing Medical University, Chongqing, 400010, China. E-mail address: [300408@hospital.cqmu.edu.cn](mailto:300408@hospital.cqmu.edu.cn).

**Figure S1.** The subgroup and interactions analyses of the association between the fT3/ fT4 ratio with all-cause mortality of MetS population.


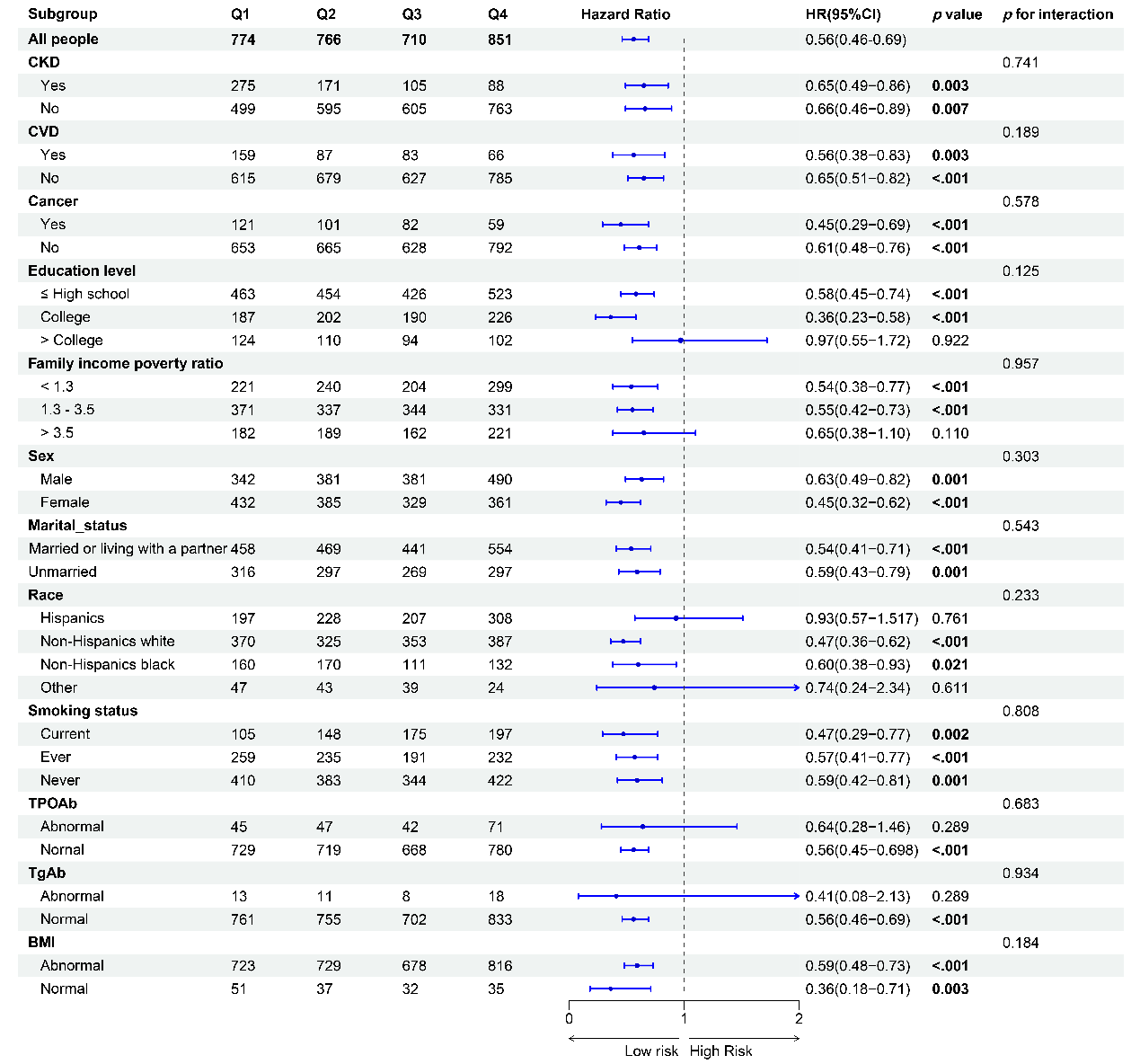


**Figure S2.** The subgroup and interactions analyses of the association between the fT3/ fT4 ratio with cardiovascular mortality of MetS population.


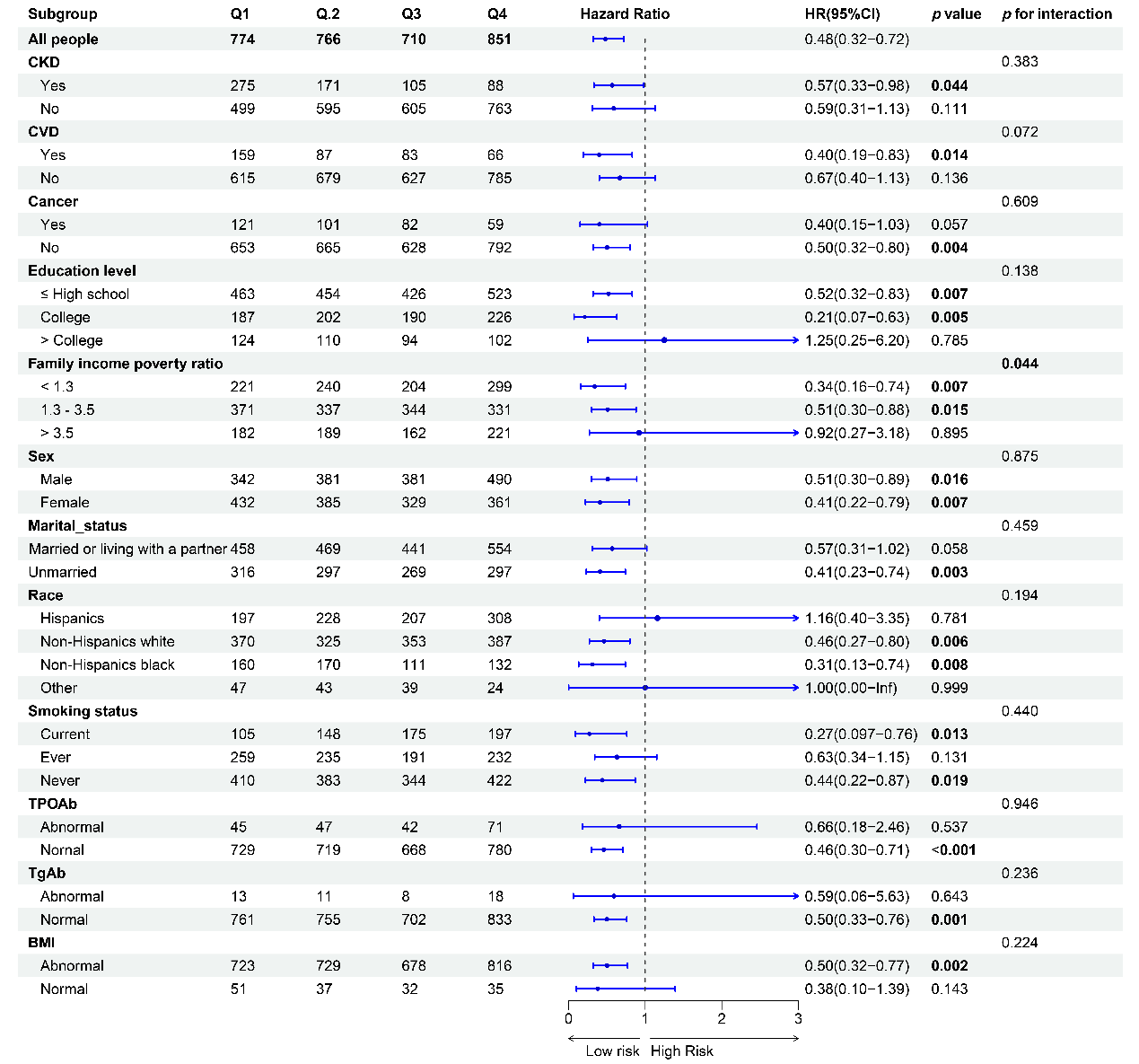


**Table S1**. The C-index of the predictive value of fT3, fT4, and fT3/ fT4 ratio in MetS population.

| **Variables** | **C-index (95%CI)** |
| --- | --- |
| **All-cause mortality** |  |
| fT3 | 0.664 (0.640 – 0.682) |
| fT4 | 0.561 (0.538 – 0.584) |
| fT3/ fT4 ratio | 0.793 (0.775 – 0.811) |
| **Cardiovascular mortality** |  |
| fT3 | 0.686 (0.640 – 0.732) |
| fT4 | 0.581 (0.531 – 0.631) |
| fT3/ fT4 ratio | 0.797 (0.761 – 0.833) |

Abbreviation: C-index: Concordance index; CI: Confidence interval.

**Table S2**. Sensitive analysis in associations between fT3/fT4 ratio with all-cause mortality of MetS population (excluded participants died within 2 years).

|  | **Model 1** |  | **Model 2** |  | **Model 3** |  |
| --- | --- | --- | --- | --- | --- | --- |
|  | **HR (95% CI)** | ***p*** | **HR (95% CI)** | ***p*** | **HR (95% CI)** | ***p*** |
| **fT3/fT4 ratio** | 0.54(0.48-0.61) | **<0.001** | 0.81(0.72-0.92) | **0.001** | 0.81(0.71-0.91) | **<0.001** |

Abbreviation: fT3: free triiodothyronine; fT4: free thyroxin; BMI: body mass index; TSH: thyroid stimulating hormone, TgAb: antithyroglobulin antibody; TPOAb: anti-thyroid peroxidase antibody; CKD: chronic kidney disease; CVD: cardiovascular disease.

Note: Model 1: unadjusted analysis; Model 2: adjusted for age, sex, and race; Model 3: adjusted for age, sex, race, marriage status, educational level, family income-poverty ratio, smoking status, alcohol use, history of cancer, chronic kidney disease, cardiovascular disease, BMI, and TSH, TgAb, and TPOAb.

**Table S3**. Sensitive analysis in associations between fT3/fT4 ratio with cardiovascular mortality of MetS population (excluded participants died within 2 years).

|  | **Model 1** |  | **Model 2** |  | **Model 3** |  |
| --- | --- | --- | --- | --- | --- | --- |
|  | **HR (95% CI)** | ***p*** | **HR (95% CI)** | ***p*** | **HR (95% CI)** | ***p*** |
| **fT3/fT4 ratio** | 0.45(0.35-0.58) | **<0.001** | 0.67(0.52-0.86) | **0.002** | 0.69(0.54-0.89) | **0.004** |

Abbreviation: fT3: free triiodothyronine; fT4: free thyroxin; BMI: body mass index; TSH: thyroid stimulating hormone, TgAb: antithyroglobulin antibody; TPOAb: anti-thyroid peroxidase antibody; CKD: chronic kidney disease; CVD: cardiovascular disease.

Note: Model 1: unadjusted analysis; Model 2: adjusted for age, sex, and race; Model 3: adjusted for age, sex, race, marriage status, educational level, family income-poverty ratio, smoking status, alcohol use, history of cancer, chronic kidney disease, cardiovascular disease, BMI, and TSH, TgAb, and TPOAb.

**Table S4**. Sensitive analysis in associations between fT3/fT4 ratio with all-cause mortality of MetS population (2007-2010).

|  | **Model 1** |  | **Model 2** |  | **Model 3** |  |
| --- | --- | --- | --- | --- | --- | --- |
|  | **HR (95% CI)** | ***p*** | **HR (95% CI)** | ***p*** | **HR (95% CI)** | ***p*** |
| **fT3/fT4 ratio** | 0.51(0.45-0.57) | **<0.001** | 0.78(0.70-0.88) | **<0.001** | 0.77(0.68-0.87) | **<0.001** |

Abbreviation: fT3: free triiodothyronine; fT4: free thyroxin; BMI: body mass index; TSH: thyroid stimulating hormone, TgAb: antithyroglobulin antibody; TPOAb: anti-thyroid peroxidase antibody; CKD: chronic kidney disease; CVD: cardiovascular disease.

Note: Model 1: unadjusted analysis; Model 2: adjusted for age, sex, and race; Model 3: adjusted for age, sex, race, marriage status, educational level, family income-poverty ratio, smoking status, alcohol use, history of cancer, chronic kidney disease, cardiovascular disease, BMI, and TSH, TgAb, and TPOAb.

**Table S5**. Sensitive analysis in associations between fT3/fT4 ratio with cardiovascular mortality of MetS population (2007-2010).

|  | **Model 1** |  | **Model 2** |  | **Model 3** |  |
| --- | --- | --- | --- | --- | --- | --- |
|  | **HR (95% CI)** | ***p*** | **HR (95% CI)** | ***p*** | **HR (95% CI)** | ***p*** |
| **fT3/fT4 ratio** | 0.44(0.34-0.56) | **<0.001** | 0.68(0.53-0.87) | **0.003** | 0.69(0.54-0.89) | **0.004** |

Abbreviation: fT3: free triiodothyronine; fT4: free thyroxin; BMI: body mass index; TSH: thyroid stimulating hormone, TgAb: antithyroglobulin antibody; TPOAb: anti-thyroid peroxidase antibody; CKD: chronic kidney disease; CVD: cardiovascular disease.

Note: Model 1: unadjusted analysis; Model 2: adjusted for age, sex, and race; Model 3: adjusted for age, sex, race, marriage status, educational level, family income-poverty ratio, smoking status, alcohol use, history of cancer, chronic kidney disease, cardiovascular disease, BMI, and TSH, TgAb, and TPOAb.

**Table S6**. Sensitive analysis in associations between fT3/fT4 ratio with cardiovascular mortality of MetS population (fT3/fT4 ratio calculated as a categorical variable, bipartite).

| **fT3/fT4 ratio** | **Model 1** |  | **Model 2** |  | **Model 3** |  |
| --- | --- | --- | --- | --- | --- | --- |
|  | **HR (95% CI)** | ***p*** | **HR (95% CI)** | ***p*** | **HR (95% CI)** | ***p*** |
| **Q1** | Reference |  | Reference |  | Reference |  |
| **Q2** | 0.48(0.40-0.56) | **0.001** | 0.81(0.68-0.96) | **0.016** | 0.79(0.66-0.94) | **0.007** |

Abbreviation: fT3: free triiodothyronine; fT4: free thyroxin; BMI: body mass index; TSH: thyroid stimulating hormone, TgAb: antithyroglobulin antibody; TPOAb: anti-thyroid peroxidase antibody; CKD: chronic kidney disease; CVD: cardiovascular disease.

Note: Model 1: unadjusted analysis; Model 2: adjusted for age, sex, and race; Model 3: adjusted for age, sex, race, marriage status, educational level, family income-poverty ratio, smoking status, alcohol use, history of cancer, chronic kidney disease, cardiovascular disease, BMI, and TSH, TgAb, and TPOAb.

**Table S7**. Sensitive analysis in associations between fT3/fT4 ratio with all-cause mortality of MetS population (fT3/fT4 ratio calculated as categorical variable, quartile).

| **fT3/fT4 ratio** | **Model 1** |  | **Model 2** |  | **Model 3** |  |
| --- | --- | --- | --- | --- | --- | --- |
|  | **HR (95% CI)** | ***p*** | **HR (95% CI)** | ***p*** | **HR (95% CI)** | ***p*** |
| **Q1** | Reference |  | Reference |  | Reference |  |
| **Q2** | 0.56(0.46-0.69) | **<0.001** | 0.75(0.61-0.92) | **0.006** | 0.75(0.61-0.92) | **0.006** |
| **Q3** | 0.43(0.35-0.54) | **<0.001** | 0.73(0.59-0.91) | **0.006** | 0.71(0.57-0.89) | **0.003** |
| **Q4** | 0.30(0.24-0.39) | **<0.001** | 0.69(0.54-0.89) | **0.004** | 0.67(0.51-0.87) | **0.002** |

Abbreviation: fT3: free triiodothyronine; fT4: free thyroxin; BMI: body mass index; TSH: thyroid stimulating hormone, TgAb: antithyroglobulin antibody; TPOAb: anti-thyroid peroxidase antibody; CKD: chronic kidney disease; CVD: cardiovascular disease.

Note: Model 1: unadjusted analysis; Model 2: adjusted for age, sex, and race; Model 3: adjusted for age, sex, race, marriage status, educational level, family income-poverty ratio, smoking status, alcohol use, history of cancer, chronic kidney disease, cardiovascular disease, BMI, and TSH, TgAb, and TPOAb.

**Table S8**. Sensitive analysis in associations between fT3/fT4 ratio with cardiovascular mortality of MetS population (fT3/fT4 ratio calculated as a categorical variable, bipartite).

| **fT3/fT4 ratio** | **Model 1** |  | **Model 2** |  | **Model 3** |  |
| --- | --- | --- | --- | --- | --- | --- |
|  | **HR (95% CI)** | ***p*** | **HR (95% CI)** | ***p*** | **HR (95% CI)** | ***p*** |
| **Q1** | Reference |  | Reference |  | Reference |  |
| **Q2** | 0.39(0.27-0.55) | **0.001** | 0.67(0.47-0.97) | **0.033** | 0.67(0.46-0.98) | **0.037** |

Abbreviation: fT3: free triiodothyronine; fT4: free thyroxin; BMI: body mass index; TSH: thyroid stimulating hormone, TgAb: antithyroglobulin antibody; TPOAb: anti-thyroid peroxidase antibody; CKD: chronic kidney disease; CVD: cardiovascular disease.

Note: Model 1: unadjusted analysis; Model 2: adjusted for age, sex, and race; Model 3: adjusted for age, sex, race, marriage status, educational level, family income-poverty ratio, smoking status, alcohol use, history of cancer, chronic kidney disease, cardiovascular disease, BMI, and TSH, TgAb, and TPOAb.

**Table S9**. Sensitive analysis in associations between fT3/fT4 ratio with cardiovascular mortality of MetS population (fT3/fT4 ratio calculated as categorical variable, quartile).

| **fT3/fT4 ratio** | **Model 1** |  | **Model 2** |  | **Model 3** |  |
| --- | --- | --- | --- | --- | --- | --- |
|  | **HR (95% CI)** | ***p*** | **HR (95% CI)** | ***p*** | **HR (95% CI)** | ***p*** |
| **Q1** | Reference |  | Reference |  | Reference |  |
| **Q2** | 0.50(0.33-0.75) | **0.001** | 0.66(0.44-0.99) | **0.044** | 0.69(0.46-1.03) | 0.070 |
| **Q3** | 0.33(0.21-0.53) | **<0.001** | 0.58(0.36-0.92) | **0.021** | 0.56(0.35-0.91) | **0.018** |
| **Q4** | 0.24(0.15-0.41) | **<0.001** | 0.55(0.33-0.93) | **0.026** | 0.59(0.34-1.02) | 0.058 |

Abbreviation: fT3: free triiodothyronine; fT4: free thyroxin; BMI: body mass index; TSH: thyroid stimulating hormone, TgAb: antithyroglobulin antibody; TPOAb: anti-thyroid peroxidase antibody; CKD: chronic kidney disease; CVD: cardiovascular disease.

Note: Model 1: unadjusted analysis; Model 2: adjusted for age, sex, and race; Model 3: adjusted for age, sex, race, marriage status, educational level, family income-poverty ratio, smoking status, alcohol use, history of cancer, chronic kidney disease, cardiovascular disease, BMI, and TSH, TgAb, and TPOAb.
